# Experimental and Theoretical Study on Minimum Achievable Foil Thickness during Asymmetric Rolling

**DOI:** 10.1371/journal.pone.0106637

**Published:** 2014-09-09

**Authors:** Delin Tang, Xianghua Liu, Meng Song, Hailiang Yu

**Affiliations:** 1 State Key Laboratory of Rolling and Automation, Northeastern University, Shenyang, China; 2 School of Mechanical, Materials & Mechatronic Engineering, University of Wollongong, Wollongong, NSW, Australia; 3 School of Mechanical Engineering, Shenyang University, Shenyang, China; Washington State University, United States of America

## Abstract

Parts produced by microforming are becoming ever smaller. Similarly, the foils required in micro-machines are becoming ever thinner. The asymmetric rolling technique is capable of producing foils that are thinner than those produced by the conventional rolling technique. The difference between asymmetric rolling and conventional rolling is the ‘cross-shear’ zone. However, the influence of the cross-shear zone on the minimum achievable foil thickness during asymmetric rolling is still uncertain. In this paper, we report experiments designed to understand this critical influencing factor on the minimum achievable thickness in asymmetric rolling. Results showed that the minimum achievable thickness of rolled foils produced by asymmetric rolling with a rolling speed ratio of 1.3 can be reduced to about 30% of that possible by conventional rolling technique. Furthermore, the minimum achievable thickness during asymmetric rolling could be correlated to the cross-shear ratio, which, in turn, could be related to the rolling speed ratio. From the experimental results, a formula to calculate the minimum achievable thickness was established, considering the parameters cross-shear ratio, friction coefficient, work roll radius, etc. in asymmetric rolling.

## Introduction

Micro-manufacturing has attracted increasing attention over recent years due to consumer-driven and industry-driven trends towards product miniaturisation in applications such as engineering and medicine [1], [2]. The global market for the microsystems technology and microelectromechanical systems reached $52 billion in 2009. The market of microelectromechanical systems is expected to grow from $11 billion in 2012 to $22.5 billion in 2018 [3]. Most of these products contain mechanical parts produced by microforming, an emerging manufacturing process that involves the fabrication of products from ultrathin foils. The foil thickness may range from 1 µm to 300 µm [4]. The growing demand on micro-manufacturing to produce smaller and smarter micro parts requires that the foils are also required to be ever thinner, while at the same time ensuring that the foils are capable of excellent mechanical performance.

Asymmetric rolling is a severe plastic deformation technique that can refine grain size in sheet/foil materials. This technique has been used to improve the mechanical properties of products [5], [6]. In the asymmetric rolling process, sheets are passed between rolls that either have different diameters, or rotate at different angular speeds. Asymmetric rolling has the potential for industrial applications because it involves a reduction in the rolling pressure and torque and an improvement in the sheet shape. It is also possible to obtain a quasi-uniform shear strain distribution across the sheet thickness under certain rolling conditions [7]. These modifications after deformation can produce significant alterations in the texture and microstructure after subsequent annealing and may thus lead to an improvement in the eventual mechanical properties of the rolled and annealed sheets [8]. In addition, the material is subjected to enhanced shear deformation. It has been suggested that high-angle boundaries develop with increasing strain, and ultrafine grains are formed by continuous recrystallization during annealing [9]. Ji and Park [10] found that the grains of magnesium alloy AZ 31 sheets were recrystallized and could be reduced to 3 µm by asymmetric rolling. Kim *et al.* [11] found the asymmetric rolling process effective in enhancing the strength of oxygen-free copper. Zou *et al.* [12] obtained a 500 nm grain-size pure aluminum sheet by asymmetric rolling. Wronski *et al.* [13] studied the grain refinement in an Al 6061 alloy by asymmetric warm-rolling. In asymmetrically rolled strips with a thickness reduction of 91.8% at 300°C, fine grains with an average size of 1 µm have been developed. It appears that during asymmetric rolling, the complete strain state imposed on the sheet is a combination of plane strain deformation and an additional shear component, which could refine the grains. Yu *et al.* [14], [15] used the asymmetric cryorolling technique to produce nanostructured Al 1050 and Al 6061 sheets. For the Al 1050 sheets, both the tensile strength and the ductility were found to increase with increase in the rolling speed ratio between the upper and lower rolls from 1.0 to 1.4.

The asymmetric rolling technique can produce thinner foils. In conventional rolling, the foils produced cannot be thinner than a certain value. When this happens, there is no method to reduce the foil thickness further. Even increasing the rolling force or the number of rolling passes does not help. In addition, the quality of products can suffer especially when the rolling mill has been operating close to its full capacity for a long time. Therefore, foil rolling becomes a technical problem when the foil is required to be thinner. This thickness limit is called the minimum achievable thickness. Two factors can influence the minimum achievable thickness: (i) **the stress state** - The region near the neutral plane of the foil is in a state of intense three-dimensional compressive stress, and plastic deformation of foil is difficult according to current plastic deformation theory; (ii) **the rolling force** – needs to be increased for thinner foils and due to work hardening. Once the mill reaches full capacity, the foils cannot be thinned any further. In the past few decades, the minimum achievable thickness of products by conventional rolling has been studied extensively [16]. Zhu *et al.* [17] reported the “elastic kernel” principle, and suggested that there was no definitely minimum achievable thickness in practical rolling process. Lian [18] studied the elastic deformation of rolls and foils. The stress and length of the elastic zone in the deformation region were calculated. A formula for precise plasticity conditions under small plastic deformation was proposed. The minimum achievable thickness in conventional rolling is a function of many factors, as shown in Eq. (1): 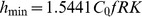
(1)where, *h*_min_ is minimum achievable thickness; *C_0_* constant related to Poisson's ratio and the Young's modulus of work roll and rolled workpiece; *ƒ* friction coefficient; *R* radius of work roll; and *K* plane deformation resistance, *K* = 1.15*σ*. The above equation suggests that the minimum achievable thickness could be reduced with reduction of friction coefficient, work roll radius, yield stress and/or by increasing the Young's modulus of the work roll material. For reducing the friction coefficients, a lubricant could be used. We could use 20-high roll mills [19]–[21] instead of 4-high roll mills to work with smaller rolls. Annealing the workpiece before rolling could lead to reduction in yield stress. Using high-speed steels and ceramic materials [22] could increase the Young's modulus of the work roll material. Compared with the conventional rolling technique, asymmetric rolling can produce thinner foils. To achieve the minimum thickness, Tzou *et al.* [23] have proposed a complete equation for asymmetric cold rolling of sheets, using an analytical approach based on the ‘slab method’. They analyzed the influence of the friction coefficient on the change in minimum thickness. However, there have been very few theoretical and experimental studies on the minimum achievable thickness in asymmetric rolling. Zhang *et al.* [24] proposed an analytical solution based on the slab method to calculate the rolling force and rolling torque in asymmetric sheet rolling. They considered cases where the work roll radii, their speeds, and the interfacial frictions may be different. Hao *et al.* [25] proposed a two-dimensional explicit dynamic finite element model using an ‘arbitrary Lagrangian-Eulerian’ adaptive meshing technique to simulate asymmetric sheet rolling, in which the asymmetry is due to different roll radii. Singh *et al.* [26] developed a formula for the prediction of minimum film thickness at the roll/strip contact in terms of operating parameters particularly at elevated roll speeds in cold strip rolling. The influence of unequal velocities of the working rolls on the reduction in the unit pressure of metal on the rolls was studied by Kawalek *et al.* [27]. They found that the magnitude of the total roll separating force can be reduced by up to 27% by introducing the asymmetric plate rolling process through different working roll peripheral speeds. Owing to the reduction of rolling load in asymmetric rolling, the minimum achievable thickness of foils is expected to be much smaller than that possible with the conventional rolling technique.

The deformation brought about by asymmetric rolling is different from that in the case of conventional rolling - Asymmetric rolling involves formation of the cross-shear zone. As seen above, some research on the mechanical properties of foils during asymmetric rolling has been carried out. However, there has been no reported research on the minimum achievable thickness of foils during asymmetric rolling. In this paper, we describe a novel method to measure and analyze the minimum achievable thickness during asymmetric rolling. The experiments were conducted using a four-high asymmetric roll mill. The effect of rolling parameters such as rolling speed ratio and cross-shear ratio on the minimum achievable thickness was analyzed in these experiments. Finally, we propose a novel theoretical model on the basis of the experimental results. The results provide a mathematical foundation for further studies of the minimum achievable thickness in asymmetric rolling.

## Experimental Investigation

### Experimental equipment

In the experiments, a four-high experimental asymmetric roll mill was employed. The main parameters of the mill are listed in **Table 1**.

**Table 1:** Parameters of experimental mill.

Mill type	Work rolling diameter [mm]	Work rolling length [mm]	Backup rolling diameter [mm]	Backup rolling length [mm]	Maximum rolling force [kN]
Ф50 Four-high asymmetric mill	50	130	120	120	200

**Figure 1:**
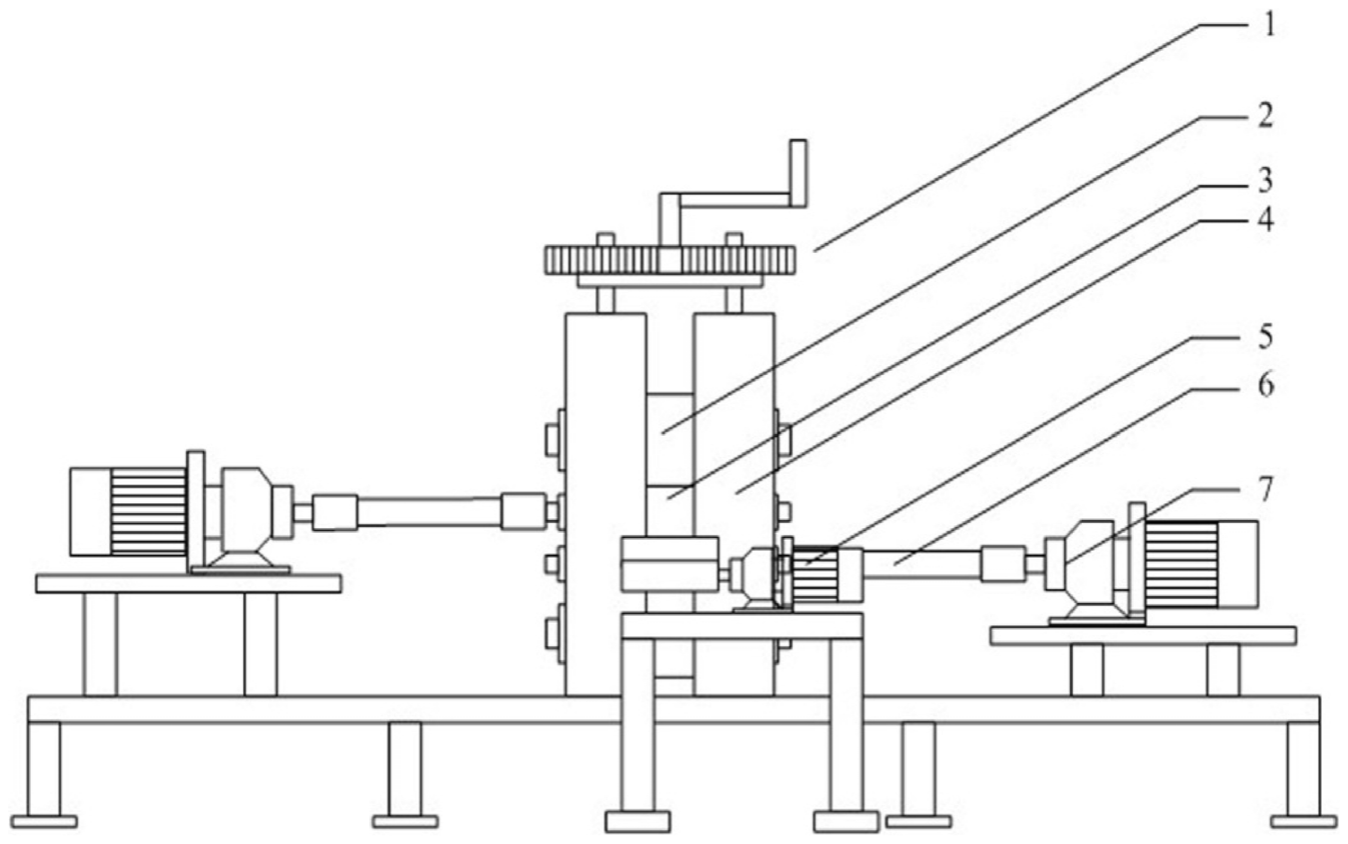
Illustration of asymmetric mill. (1. Screw-down device; 2.Backup roll; 3.Work roll; 4.mill house; 5.Tensile motor; 6.Universal shaft; 7. Main drive motor).

**Figure 1** illustrates the Ф50 four-high asymmetric mill. The transmission shafts are on either side of the mill. The rolling speed of the upper and lower work rolls could be independently adjusted to meet the requirement in the experiments. Here, we define the rolling speed ratio as the ratio of the upper roll speed to the lower roll speed. In this roll mill, the rolling speed ratio can be set at any value. The speed of the motor can be recorded by an incremental encoder installed at the rear of the motor, and then the roll speed can be calculated on the basis of the motor speed. The rolling force can be obtained by pressure sensors. The maximum rolling force of the mill is of 200 kN.

**Figure 2:**
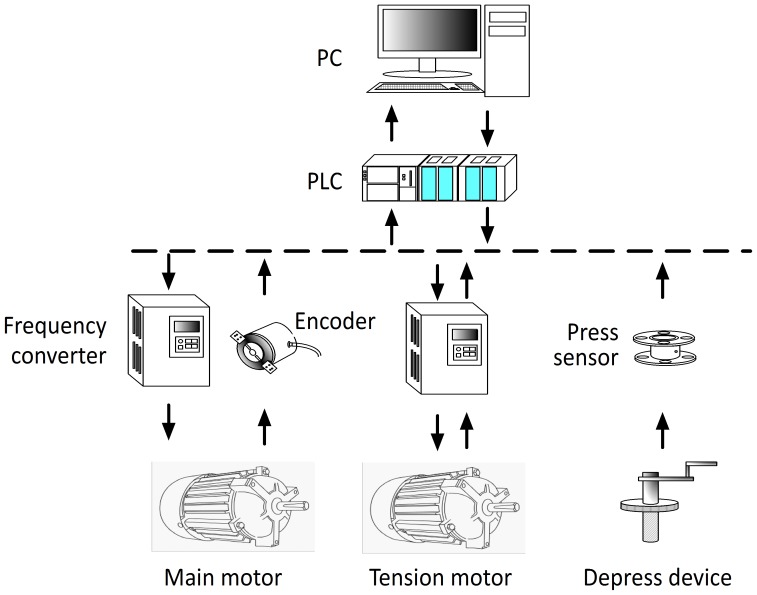
Illustration of control system.

**Figure 2** shows the control system of the asymmetric mill, consisting of a programmable logic controller control console, human-machine interface and rolling parameters detection device. Rolling parameters such as rolling speed, tension force and asymmetry ratio can be set on the human-machine interface and the information is passed to the programmable logic controller program to control the roll mill. The detected data in the rolling process are also shown on the human-machine interface by programmable logic controller program.

### Experimental schedules

The roll mill described above was used in the experiments. The employed coils were made of Q195 steel, whose composition is listed in **Table 2**.

**Table 2:** Chemical composition of Q195.

Q195	C	Mn	Si	S	P	Fe
w%	≤0.12	≤0.50	≤0.30	≤0.040	≤0.035	Balance

In the experiments, multi-pass rolling was carried out until the foils were rolled to the minimum achievable thickness. In order to avoid the influence of other parameters on the minimum achievable thickness, only the rolling speed ratio was changed, with the materials, friction condition, work rolls kept fixed. Samples with initial thickness 0.35 mm were rolled to the minimum achievable thickness with rolling speed ratios of 1.0, 1.1, 1.2 and 1.3. All rolling experiments were without application of tension. The experiments would not be stopped until the thicknesses of foils were unchanged for the last three passes, for all four rolling speed ratios. After each pass, the thickness of rolled sheets was measured with a micrometer at 3 different points. The average thickness value after each pass was recorded, in addition to the linear velocities of work rolls, exit thickness of foil and exit velocity in each rolling pass. The roll speed ratio was a vital parameter for adjusting the cross-shear ratio.

### Cross-shear ratio (*ω*)

**Figure 3:**
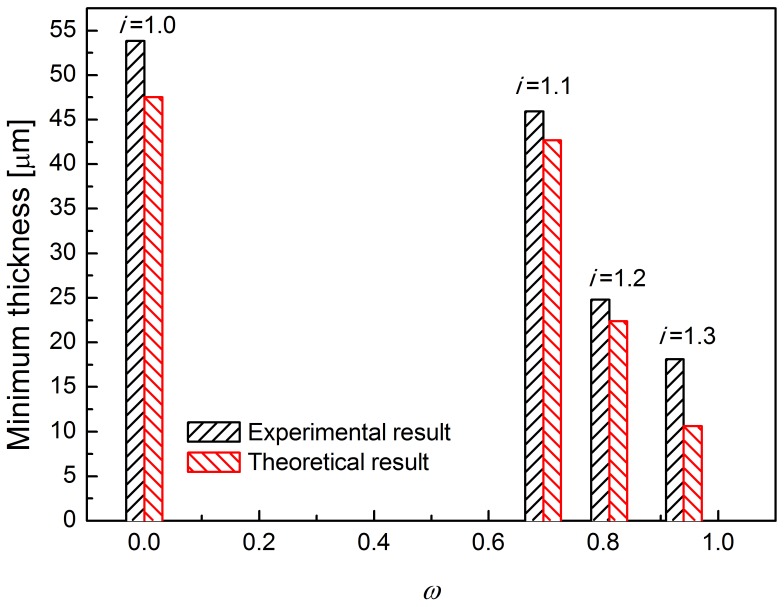
Illustration of deformation region, (a) conventional rolling, (b) low rolling speed ratio in asymmetric rolling, (c) high rolling speed ratio in asymmetric rolling.

The cross-shear ratio is the ratio of the area of the cross-shear zone to the area of the whole plastic deformation region, as shown in **Figure 3** [14]. It is obvious that the difference between conventional rolling and asymmetric rolling is the appearance of the cross-shear zone. We focused on an analysis of the influence of the cross-shear ratio on the minimum achievable thickness, which is related to the plastic deformation configuration, rolling speed ratio, exit velocity, exit thickness and linear rolling velocity. In the rolling process, the deformation region is generally divided into three parts. In conventional rolling (*V_1_* = *V_2_*), as shown in Figure 3(a), the deformation region contains a forward-slip zone and a backward-slip zone. In asymmetric rolling (*V_1_>V_2_*), as shown in Figure 3(b), the deformation region contains a forward-slip zone, a cross-shear zone and a backward-slip zone. When the rolling speed ratio is very high (*V_1_>>V_2_*), the deformation region consists of only the cross-shear zone, as shown in Figure 3(c). In this study, we consider the rolling speed ratio up to 1.3, which can be regarded as a low rolling speed ratio.

**Figure 4:**
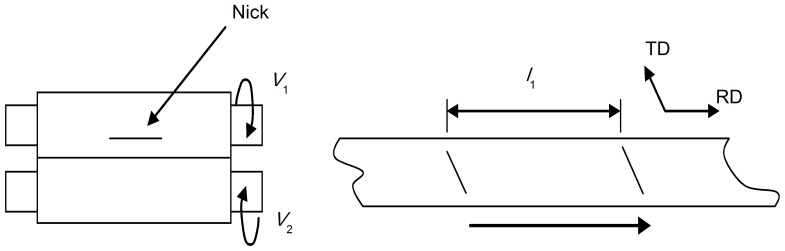
Illustration of measuring the speed of sheet during asymmetric rolling.

We used the ‘nick’ method to measure the entrance and exit speeds of the sheet, as shown in **Figure 4**. In the experiments, the value of rolling speed (*V_i_*), marked length (*l*_1_), work roll radius (*R*), foil thickness before (*H*) and after rolling (*h*), the existence and entrance speeds of sheets are shown in Eq. (2) and (3).
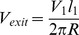
(2)

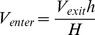
(3)where, *V_entry_* is entry speed of foil and *V_exit_* is exit speed of foil. From the geometry of the deformation region, it is possible to calculate the cross-shear ratio. The ways to calculate cross-shear ratio in different deformation region types differ because of their different geometrical shape. The cross-shear zone is zero in conventional rolling, and it is 1 for the high rolling speed ratio asymmetric rolling. For intermediate speed ratios in asymmetric rolling, the cross-shear ratio can be shown to be:
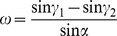
(4)


In order to calculate the cross-shear ratio, it is important to calculate the angles *γ_1_, γ_2_* and *α*.

As shown in Figure 3 (b), *α* can be calculated from the biting angle formula:
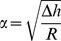
(5)


The influence of elastic deformation of work rolls on the biting angle in the rolling deformation zone is ignored.

In order to calculate *γ*_1_ and *γ*_2_, we make the following assumptions:

1) The friction coefficients between the strip and the work rolls are same and constant; and the rolling pressure is uniform in the rolling deformation zone [28].

2) The cumulative stress along the rolling direction is zero, so that:
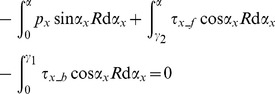
(6)


In the rolling deformation zone,
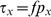
(7)


Thus, equation (6) can be written as,
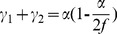
(8)where, *τ_x_b_* is shear stress at X position over the backward zones; *τ_x_f_* shear stress at X position over the forward zones; *τ_x_* shear stress at X position over the entire deformation region; and *p_x_* is average unit rolling force at X position over the entire deformation region.

**Figure 5:**
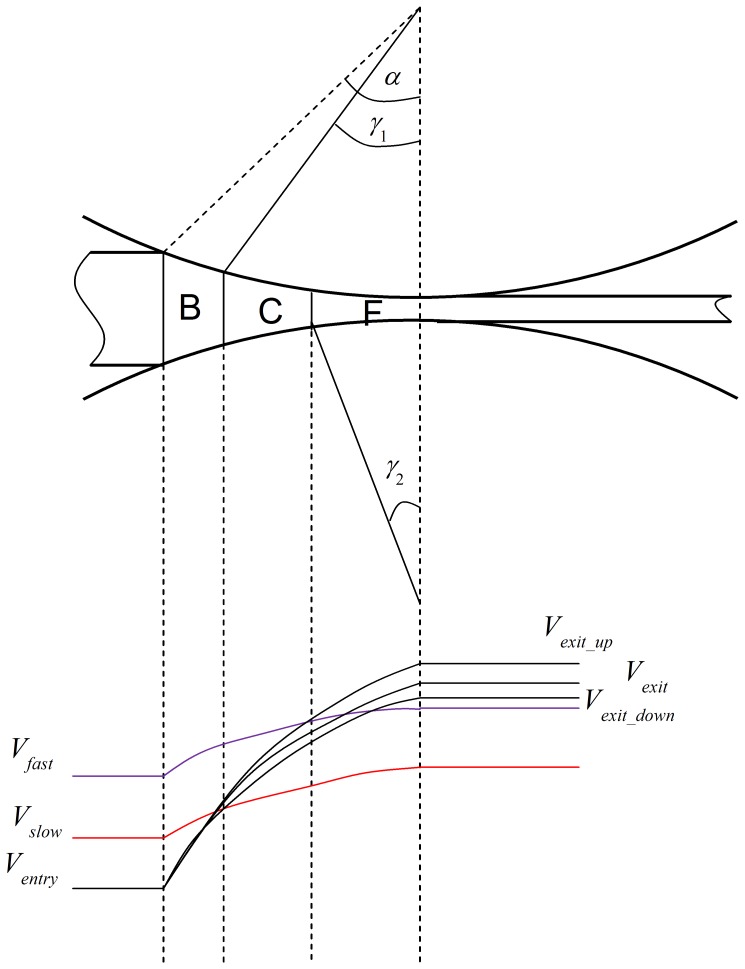
Illustration of rolling deformation zone.

3) Due to the very small thickness of foils, here we assumed that the mean rolling velocity nearly equals that at the neural surfaces, according to the principle of equal flow at two neutral surfaces. Thus, assuming that the mean rolling speed at neural surface of *γ*_1_ equals the surface speed of the upper roll, and the mean rolling speed at neural surface of *γ*_2_ equals the surface speed of lower roll. Thus, referring to **Figure 5**, when the rolling deformation zone contains a backward slip zone, a cross-shear zone and a forward slip zone, 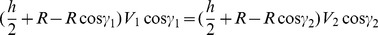
(9)


If there only the backward slip zone and the cross-shear zone exist in the rolling deformation zone, Eq. (9) can be simplified as:
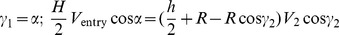
(10)


If there are only the cross-shear zone and the forward slip zone in the rolling deformation zone, Eq. (9) can be simplified as:
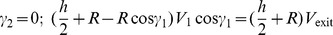
(11)


Lastly, if only the cross-shear zone exists in the rolling deformation zone, Eq. (9) reduces to:
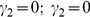
(12)


In the experiment, the *V*_1_, *V*_2_, *V_entry_*, *V_exit-up_* and *V_exit-down_* could be measured. Based on the Eqs (8)–(12), the *γ*_1_ and *γ*_2_ could be obtained. The cross-shear ratio could be obtained from Eqs (4) and (5).

## Results

**Figure 6:**
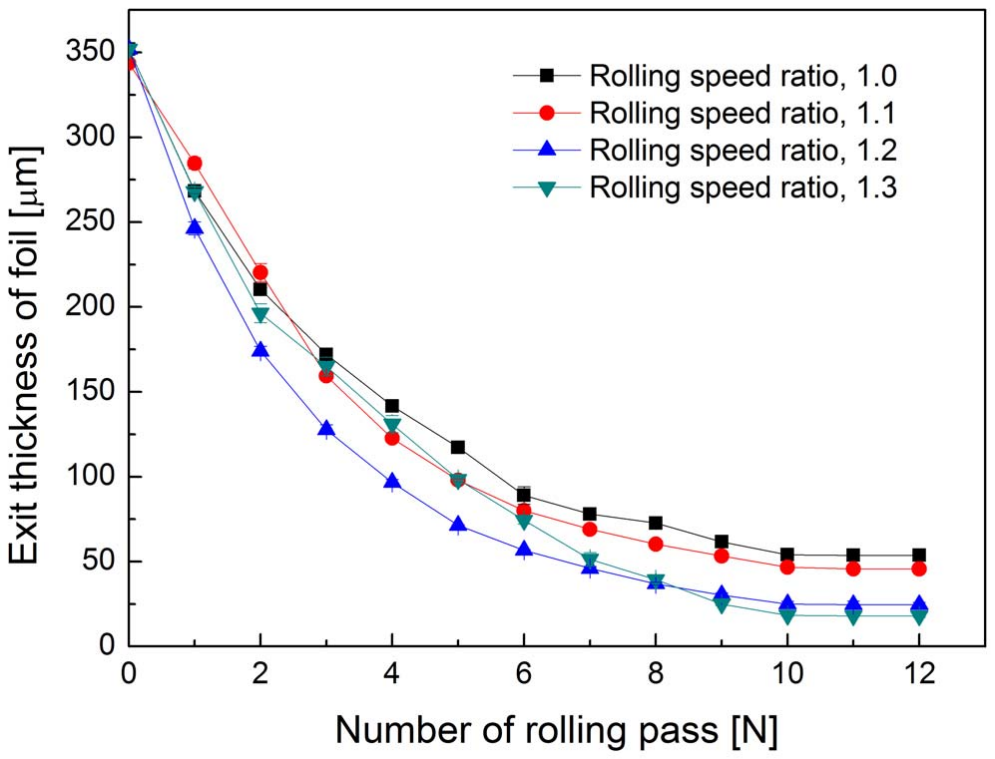
Thickness of foils for asymmetric rolling under different rolling speed ratios.

**Figure 7:**
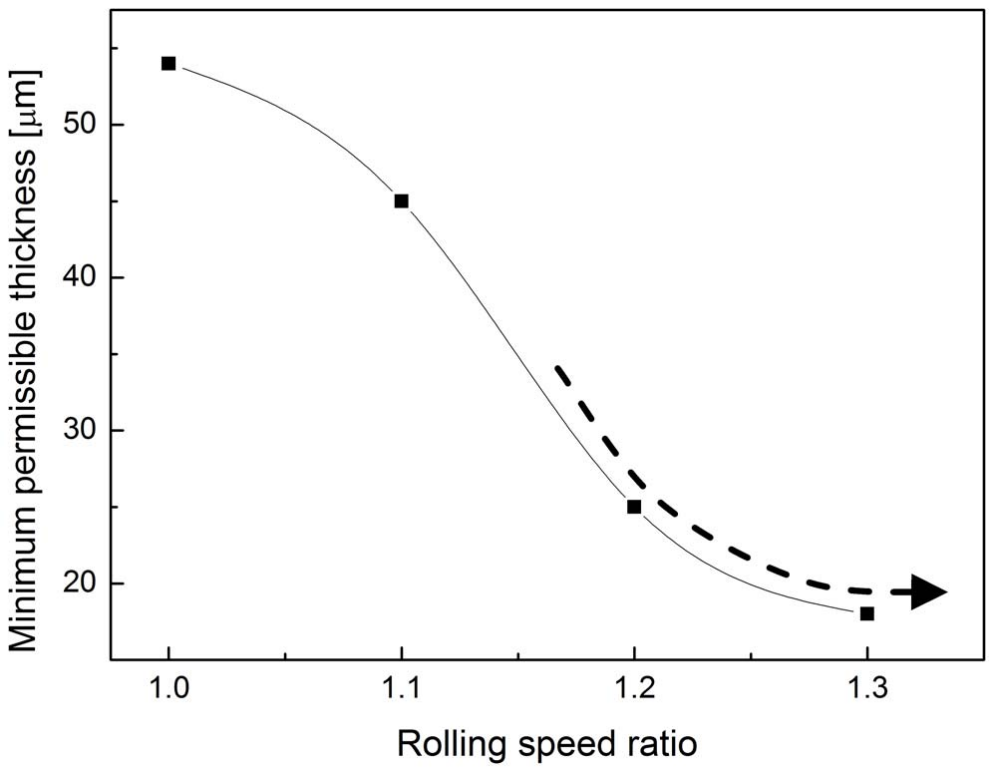
Minimum achievable thickness for asymmetric rolling for different rolling speed ratios.

**Figure 6** shows the thickness of the foils after each pass, at rolling speed ratios 1.0, 1.1, 1.2 and 1.3. The higher the rolling speed ratio, the faster the reduction in foil thickness. When the rolling speed ratio (*i*) is set as 1.0 (conventional rolling), the minimum achievable thickness was significantly larger than that by asymmetric rolling, which is 54 µm. During asymmetric rolling, the minimum achievable foil thickness gradually decreases to 18 µm with an increase in the rolling speed ratios to 1.3. The minimum thickness achievable by asymmetric rolling is only 30% of that possible by conventional rolling. **Table 3** lists the foil thickness after the final three passes. When the rolling speed ratio is 1.0, the thickness is about 54 µm. When the rolling speed ratio increases into 1.1, the thickness is reduced to 46 µm. When the rolling speed ratio increases to 1.2, the thickness is further reduced to 25 µm. When the rolling speed ratio further increases to 1.3, the thickness is reduced to 18 µm. The minimum achievable thicknesses of foils under different rolling speed ratios are shown in **Figure 7**. It appears that the minimum achievable foil thickness of foils may decrease slightly for higher rolling speed ratios.

**Table 3:** Foil thickness at the final pass under various rolling speed ratios [µm].

Rolling pass	1.0		1.1		1.2		1.3	
	Thickness	Error	Thickness	Error	Thickness	Error	Thickness	Error
10	54.0	0.8	46.7	1.2	25.0	1.6	18.3	0.4
11	53.7	1.2	45.7	0.4	24.7	2.0	18.0	0.0
12	53.7	0.4	45.7	0.4	24.7	1.2	18.0	0.8

**Figure 8:**
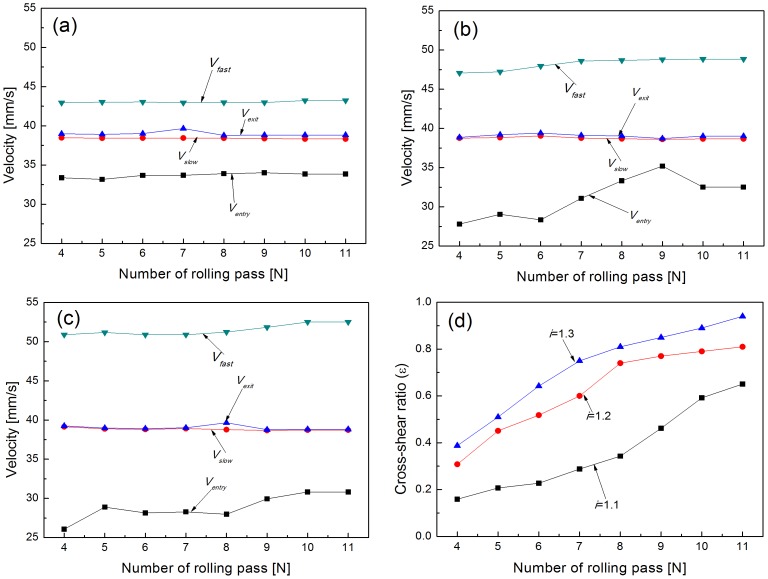
Speed of foil and rolls for (a) *i* = 1.1, (b) *i* = 1.2, (c) *i* = 1.3, and (d) cross-shear ratio from the 4th to 11th rolling pass.

As described before, in asymmetric rolling, the cross-shear ratio was a vital parameter related to the rolling speed ratio. Three groups of parameters such as entrance and exit velocities, entrance and exit thicknesses and the roll velocities were recorded from the 4th pass to the 11th pass. These were used to calculate the cross-shear ratio in the rolling process. **Figure 8** shows the rolling speeds and calculated cross-shear ratios under various rolling speed ratios. The parameters corresponding to rolling speed ratio *i* = 1.1 are listed in **Table 4**. It can be seen that the entry velocity increased significantly while the exit velocity deceased gradually as the foil got progressively thinner. The cross-shear ratio increased gradually as the number of rolling passes increased. The cross-shear ratio reached a maximum value when the foil reached the minimum achievable thickness. **Table 5** shows the minimum and maximum of cross-shear ratio with different rolling speed ratios. It can be seen that the minimum and maximum cross-shear ratio increased as the rolling speed ratio increased. The rolling deformation zone is nearly made up of only the cross-shear zone that the maximum cross-shear ratio was 0.94 while the rolling speed ratio was 1.3.

**Table 4:** Calculation results of experimental data and cross-shear ratio in each pass for rolling speed ratio 1.1.

Rolling pass	Slow roll velocity [mm/s]	Fast roll velocity [mm/s]	Exit thickness [µm]	Entry velocity [mm/s]	*V_exit-up_* [mm/s]	*V_exit-down_* [mm/s]	Cross-shear ratio
0	39.4	43.4	343.6	-	-	-	
1	38.7	42.9	284.6	32.0	39.3	43.5	0.159
2	38.6	42.9	220.3	30.9	39.3	46.2	0.207
3	38.4	42.9	159.3	32.6	39.4	45.4	0.227
4	38.4	43.0	122.6	41.2	47.8	55.5	0.285
5	38.4	43.0	98.0	34.2	38.7	44.9	0.287
6	38.4	42.9	80.0	36.0	39.0	44.6	0.288
7	38.4	42.9	69.0	36.4	38.7	44.9	0.462
8	38.3	42.9	60.0	36.9	38.7	44.3	0.592
9	38.3	43.2	53.3	36.4	38.4	44.9	0.651
10	38.2	43.3	46.6	40.9	38.5	45.0	0.695
11	38.2	43.3	45.6	41.9	38.5	45.3	0.695
12	38.2	43.2	45.6	41.5	38.5	45.3	0.695

**Table 5:** Cross-shear ratio in different rolling speed ratio at the 11th pass.

Rolling speed ratio	1.0	1.1	1.2	1.3
Cross-shear ratio for 11th rolling pass (*ω*)	0	0.695	0.810	0.94

**Figure 9:**
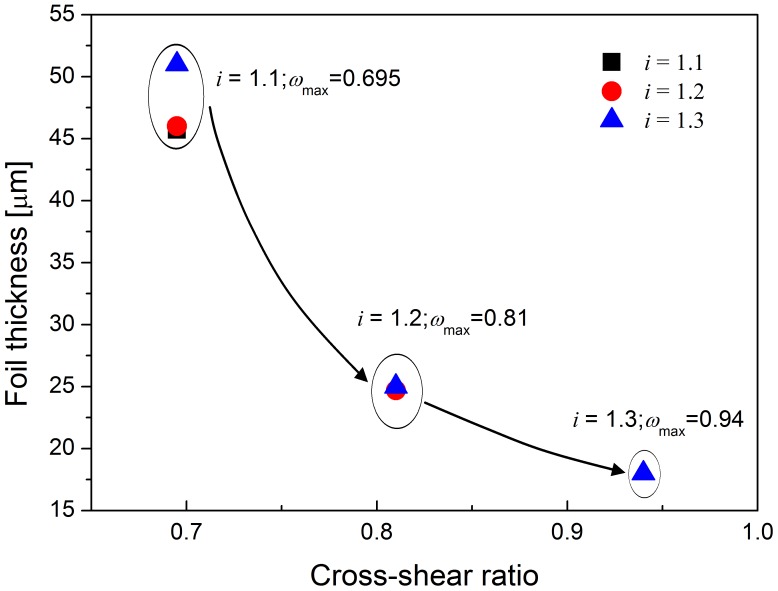
Relationship between cross-shear ratio and exit thickness reduction.

**Figure 9** shows the relationship between the maximum cross-shear ratio and minimum achievable thickness during asymmetric rolling. When the rolling speed ratio is 1.1, the maximum cross-shear ratio reaches 0.695, and the minimum achievable thickness of rolled foil is about 46 µm, which is similar to that when the cross-shear ratio at such value for rolling speed ratio 1.2 and 1.3. When the rolling speed ratio is 1.2, the maximum cross-shear ratio reaches 0.81 and the minimum achievable thickness of foil is 24.7 µm, which is nearly the same value 25 µm with the similar cross-shear zone when the rolling speed ratio is 1.3. When the rolling speed ratio increases to 1.3, the maximum cross-shear ratio reaches 0.94, and the minimum achievable thickness of foil is reduced to 18 µm. From the figure, it is obvious that the minimum achievable thickness of foil during asymmetric rolling is directly related to the maximum cross-shear ratio during asymmetric rolling.

## Discussion

In conventional rolling, the minimum achievable thickness is approximately proportional to the diameter of the work rolls and the deformation resistance of the sample material. However, in asymmetric rolling process, the minimum achievable thickness is also affected by the rolling speed ratio and the cross-shear ratio. It is seen in **Figure 7** that the minimum achievable thickness is reduced from 54 µm to 18 µm when using the asymmetric rolling with the rolling speed ratio is 1.3.

In asymmetric rolling, the difference of linear velocities of the two work rolls leads to the plastic deformation region being divided into three parts: (1) the forward-slip zone, (2) the cross-shear zone and (3) the backward-slip zone, as shown in **Figure 3**. The frictional force at the upper and lower surfaces of the foil is in the same direction, towards the cross-shear zone in both the forward-slip zone and the backward-slip zone. The velocity of the foil is the average of the linear velocities of the slower and faster work rolls in the cross-shear zone. Therefore the frictional force on the foil surface on the side of the faster work roll acts towards the backward-slip zone, while the frictional force on the foil surface on the side of the slower work roll acts towards the forward-slip zone. This results in an asymmetry in the operative frictional forces. In the following paragraphs, we propose a formula to calculate the minimum achievable thickness, based on an analysis of strain state in the deformation region.

To simplify the formulation involved in the analysis, the following assumptions and simplifications are made:

(1) The rolling process is approximately one of flat compression;

(2) The plastic deformation is a state of plane strain;

(3) Friction forces on the contact surfaces are given by Coulomb's law of friction.

(4) The tension forces at the entrance and exit are equal in magnitude, which ensures that the length of the forward-slip zone is equal to the length of backward-slip zone.

**Figure 10:**
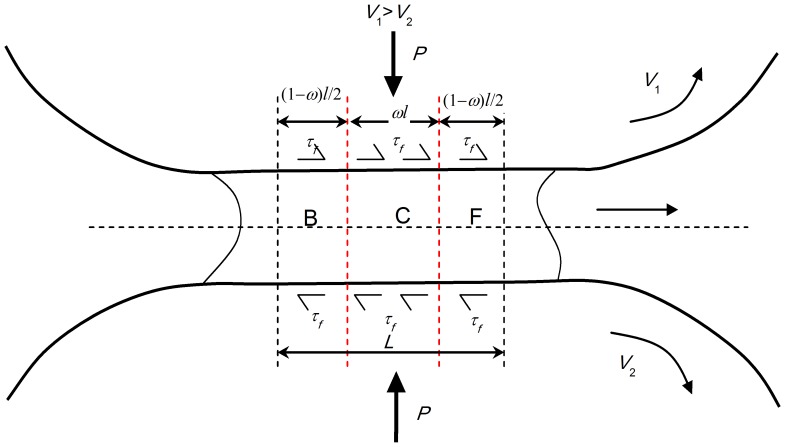
Deformation region based on Stone's assumptions.

As shown in **Figure 10**, the deformation region is divided into three parts. The arc length is *l*. Length of cross-shear zone is *ωl = l_1_+l_2_*. Length of forward-slip zone is *l/2-l_2_*; Length of backward-slip zone is *l/2-l_1_*.

According to the Stone formula [29], the unit average rolling force in the forward-slip zone is:
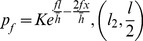
(13)


The unit average rolling force in the cross-shear zone:
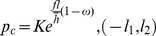
(14)


The unit average rolling force in the backward-slip zone:
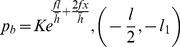
(15)


Note that only the value of the cross-shear ratio was considered. So it is assumed that *l_1_ = l_2_ = l/4*. Then the average rolling force of asymmetric rolling can be derived as: 
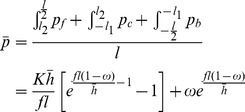
(16)


Considering elastic deformation of the foil and rolls, the Hitchcock equation [30] is as follows:
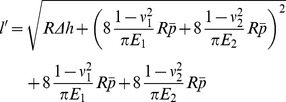
(17)


Let 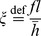, 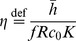, 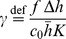,

and 
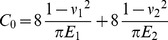
,

Then,
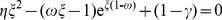
(18)where, *p_f_* is average unit rolling force in the forward-slip zone; *p_c_* average unit rolling force in the cross-shear zone; *p_b_* average unit rolling force in the backward-slip zone; 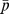 average rolling force; 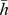 average thickness from entrance to exit; *E*_1_ and *E*_2_ Young's modulus of workpiece and work roll material respectively; Δ*h* difference in foil thickness of entrance and exit; *l* arc length of contact; 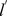 arc length of contact considering elastic deformation of foil and roll; *P* rolling force; *σ* tensile stress of workpiece material; *ν*_1_ and *ν*_2_ are Poisson ratio of workpiece and work roll materials respectively.

If Eq. (18) has a positive root, and the *η* can be calculated while for a constant *γ*. Consider Eq. (18) as an equation relating *η* and *ξ*. When *η* approach the extreme value *η_c_*, the relationship between *η* and *ξ* can be calculated using d*η*/d*ξ* = 0, then:
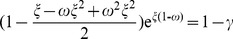
(19)


**Figure 11:**
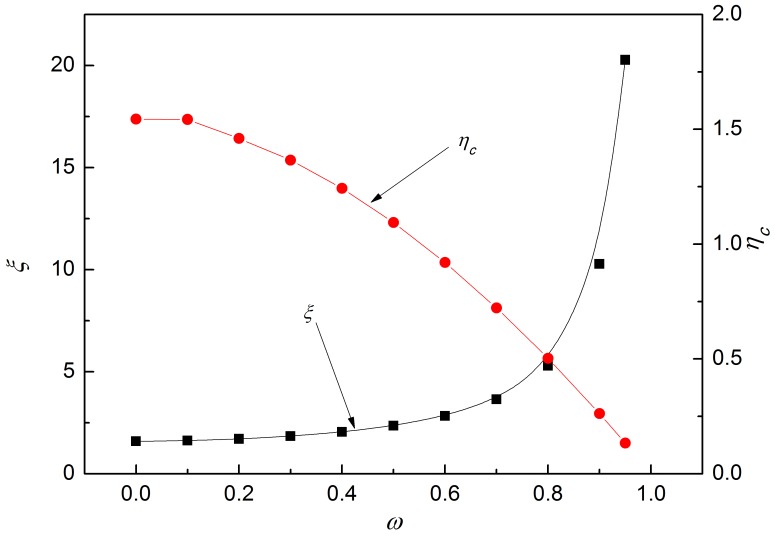
*η_c_* and *ξ* vs *ω*.

When *η* = *η_c_*, then *γ* = 1. The *ξ* value is determined solely by the *ω* value. And the *η_c_* can be calculated from *ξ* and *ω* according to Eq. (18). The relation between *η_c_* and *ω* is shown at **Figure 11**.

In **Figure 11**, the *η_c_* decreases with cross-shear ratio (*ω*) while *ξ* increases with *ω*. When *ω* = 0, *η_c_* = 1.5441 and *ξ* = 1.5936. That explains why the minimum achievable thickness decreased with cross-shear ratio which increases with the rolling speed ratio shown as in **Figure 9**.

Eq. (19) has a unique solution when *ξ_c_*>0. The extreme value *η_c_* of *η* can be solved from Eq. (19). The minimum achievable thickness can be described as:
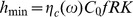
(20)


In asymmetric rolling, *η_c_* ranges from 0^+^ to 1.5441 as *ε* ranges from 0^+^ to 1. When *ω* = 0, *η_c_* = 1.5441. The minimum achievable thickness with conventional rolling can be estimated using Eq. (1). Eq. (1) fits well with the formula given by Keller under conventional rolling conditions. Eq. (1) shows that the minimum thickness of the foil achievable in conventional rolling is independent of the reduction in foil thickness in each pass. Nevertheless, analysis shows that the minimum foil thickness possible in conventional rolling is proportional to the deformation resistance of the foil and the diameters of the work rolls. This agrees well with the experimental results.

**Figure 12:**
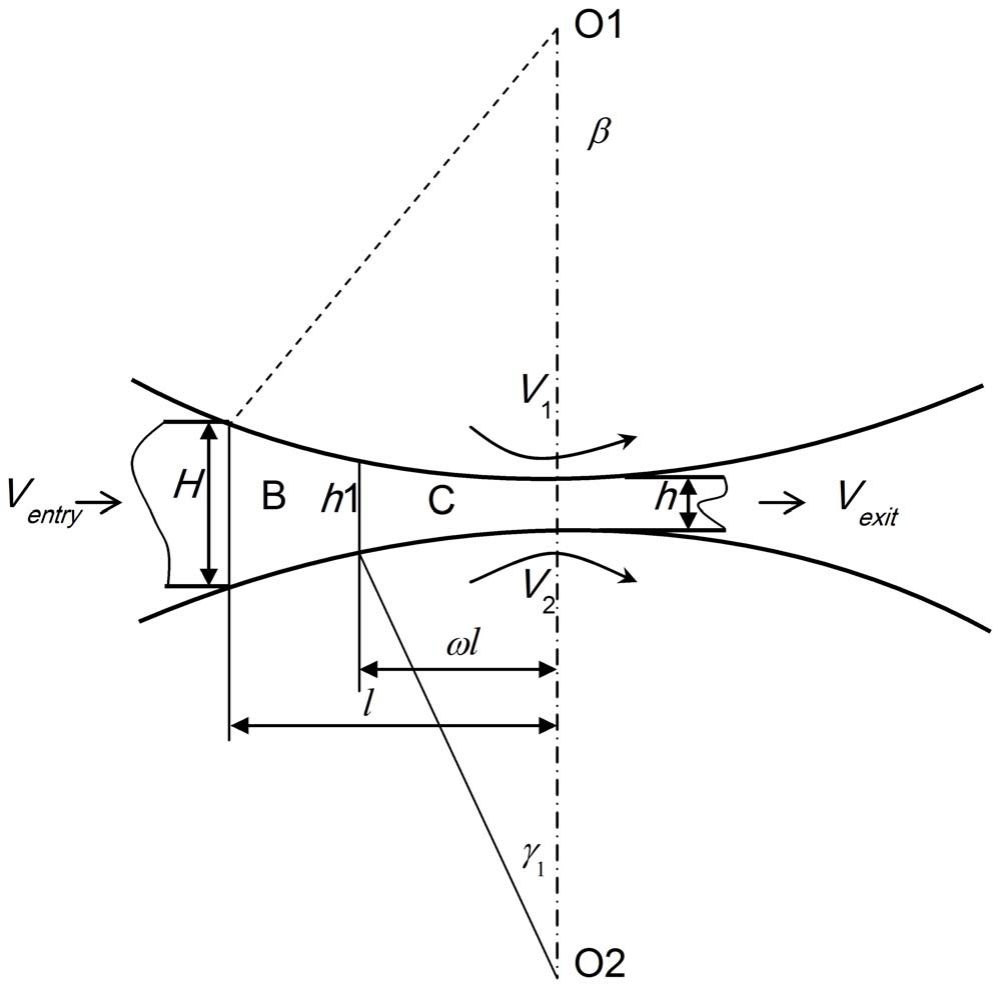
Deformation regions under experimental conditions.

**Figure 12** shows the typical parameters related to cross-shear ratio in the rolling experiment with *i* = 1.1. The deformation region is made up of the backward slip zone and the cross-shear zone.

From the geometry, *ω* can be calculated using Eq. (4) and Eq.(10). Then cos*γ*_1_ can be calculated because *h*, *R*, *V*_1_, *V*_2_ and *V_exit_* are already known:
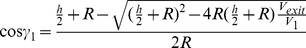
(21)

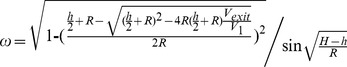
(22)


In **Figure 11**, we could fit the relationship between 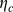 and *ω*

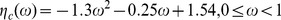
(23)


Thus, the minimum achievable thickness by asymmetric rolling could be transferred into Eq. (24) from Eq. (20). 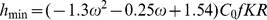
(24)


Eq. (24) shows that the minimum achievable thickness of foil in asymmetric rolling overcomes the limitations imposed in conventional rolling, due to the creation of the cross-shear zone. The minimum thickness of foil in asymmetric rolling is a function of the cross-shear ratio, the coefficient of friction between the foil and the rolls, the diameters of the work rolls and the deformation resistance of the foil. As the rolling speed ratio increases, the cross-shear zone increases in size, leading to greater foil reduction during the pass, and consequently the minimum thickness decreases.

**Figure 13:**
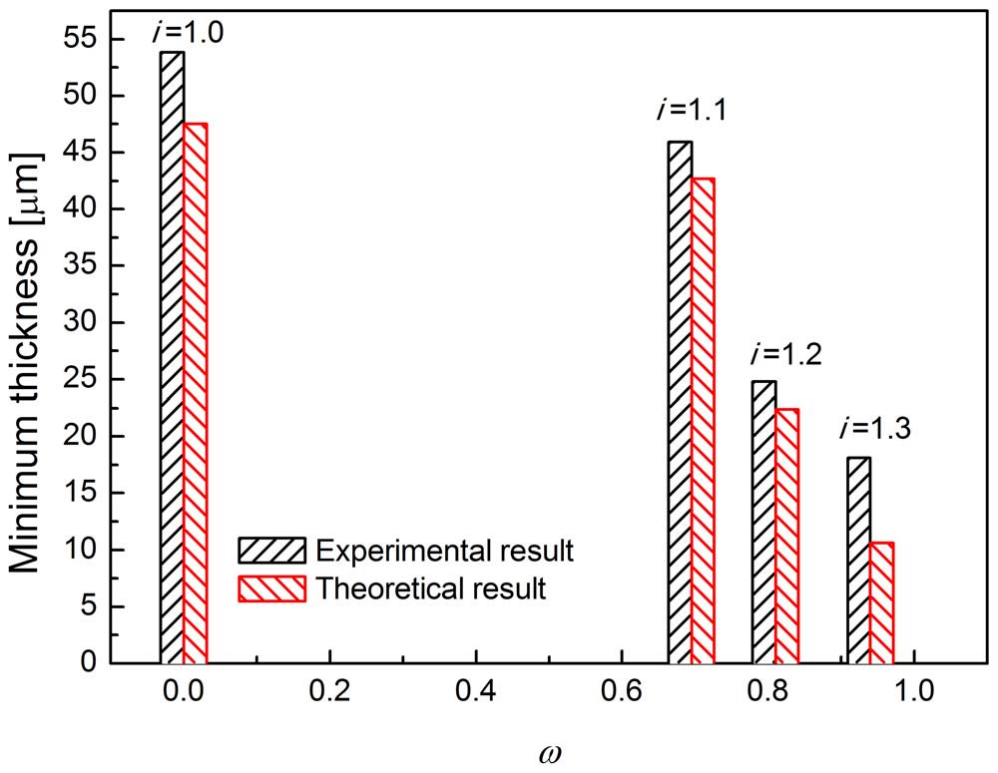
Theoretical and experimental results for various rolling speed ratios.

Eq. (24) can predict the minimum achievable foil thickness during asymmetric rolling. Based on the experiment, *ƒ* = 0.15; *C*_0_ = 2.3×10^−11^; the yield strengths estimated by tensile tests are 230 MPa, 240 MPa, 249.6 MPa and 260.9 MPa for the last exit thicknesses for rolling speed ratios 1.0, 1.1, 1.2 and 1.3, thus the values of *K* (*K* = 1.15*σ*) are 240 MPa, 260 MPa, 287 MPa and 300 MPa respectively; *R* = 24.3 mm (actual work roll radius). *ω* is listed in **Table 5**. **Figure 13** compares the theoretical and experimental results, showing that the theoretical minimum achievable foil thicknesses are smaller than the experimental values. When *i* = 1.0, the theoretical minimum achievable foil thickness can reach 47.5 µm, however, experimentally, only 53.8 µm is achievable. When *i* = 1.1 and *i* = 1.2, the theoretical and experimental results differ by 3.2 µm and 2.4 µm respectively. For *i* = 1.3, the error increases into 7.5 µm. The difference between the theoretical and experimental results appears to be due to the rolling load of the mill in the experiments. Generally, the theoretical values represent ideal limits, which will be slightly less than the experimental ones owing to the experimental conditions. In the theoretical model, the rolling force is assumed to be infinite. Before the thickness of the foils is reduced into the minimum thickness, the rolling force will continuously increase. However, in this experiment, the rolling load of mill is limited to a maximum value of 200 kN. The rolling force provided by the mill is not high enough to roll the foils to the theoretical minimum achievable thickness. In order to achieve a thickness close to the theoretical minimum, a mill of higher rolling load should be used. But, it is obvious that the additional parameter, viz. the cross-shear zone ratio, Eq. (21), is an effective parameter to predict the minimum achievable thickness during asymmetric rolling.

## Summary

Experimental results show that the minimum achievable thickness achievable by asymmetric rolling with the rolling speed ratio 1.3 is 30% of that possible by conventional rolling.

A new formula, Eq. (24), has been developed to predict the minimum achievable thickness (*h_min_*) during asymmetric rolling. The minimum achievable foil thickness is shown to be a function of the cross-shear ratio, friction coefficient, deformation resistance, work roll radius, Young's modulus of work roll. As the rolling speed ratio increases, the cross-shear ratio increases and the minimum achievable thickness decreases.

The cross-shear ratio is related to the rolling speed ratio, the entry and exit speeds of the foil and the linear speed of the upper and lower rolls. When the deformation region is made up of three parts, the cross-shear ratio can be calculated as shown in Eq. (22). As the foil thickness decreases, the exit speed of the foil tends to the linear speed of the slower work roll and the cross-shear ratio increases in multi-pass asymmetric rolling.
